# An end-to-end multi-task system of automatic lesion detection and anatomical localization in whole-body bone scintigraphy by deep learning

**DOI:** 10.1093/bioinformatics/btac753

**Published:** 2022-11-23

**Authors:** Kaibin Huang, Shengyun Huang, Guojing Chen, Xue Li, Shawn Li, Ying Liang, Yi Gao

**Affiliations:** School of Biomedical Engineering, Health Science Center, Shenzhen University, Shenzhen 518037, China; National Cancer Center/National Clinical Research Center for Cancer/Cancer Hospital & Shenzhen Hospital, Chinese Academy of Medical Sciences and Peking Union Medical College, Shenzhen 518116, China; School of Biomedical Engineering, Health Science Center, Shenzhen University, Shenzhen 518037, China; School of Biomedical Engineering, Health Science Center, Shenzhen University, Shenzhen 518037, China; School of Biomedical Engineering, Health Science Center, Shenzhen University, Shenzhen 518037, China; National Cancer Center/National Clinical Research Center for Cancer/Cancer Hospital & Shenzhen Hospital, Chinese Academy of Medical Sciences and Peking Union Medical College, Shenzhen 518116, China; National Cancer Center/National Clinical Research Center for Cancer/Cancer Hospital, Chinese Academy of Medical Sciences and Peking Union Medical College, Beijing 100021, China; School of Biomedical Engineering, Health Science Center, Shenzhen University, Shenzhen 518037, China; Shenzhen Key Laboratory of Precision Medicine for Hematological Malignancies, Shenzhen 518037, China; Marshall Laboratory of Biomedical Engineering, Shenzhen 518037, China

## Abstract

**Summary:**

Limited by spatial resolution and visual contrast, bone scintigraphy interpretation is susceptible to subjective factors, which considerably affects the accuracy and repeatability of lesion detection and anatomical localization. In this work, we design and implement an end-to-end multi-task deep learning model to perform automatic lesion detection and anatomical localization in whole-body bone scintigraphy. A total of 617 whole-body bone scintigraphy cases including anterior and posterior views were retrospectively analyzed. The proposed semi-supervised model consists of two task flows. The first one, the lesion segmentation flow, received image patches and was trained in a supervised way. The other one, skeleton segmentation flow, was trained on as few as five labeled images in conjunction with the multi-atlas approach, in a semi-supervised way. The two flows joint in their encoder layers so each flow can capture more generalized distribution of the sample space and extract more abstract deep features. The experimental results show that the architecture achieved the highest precision in the finest bone segmentation task in both anterior and posterior images of whole-body scintigraphy. Such an end-to-end approach with very few manual annotation requirement would be suitable for algorithm deployment. Moreover, the proposed approach reliably balances unsupervised labels construction and supervised learning, providing useful insight for weakly labeled image analysis.

**Supplementary information:**

Supplementary data are available at *Bioinformatics* online.

## 1 Introduction

Bone scintigraphy has been one of the most commonly performed examinations in routine nuclear medicine practice. It is useful for suspected bone metastases in a number of cancers and for the investigation of many benign skeletal conditions because of its sensitivity, availability, low cost and large field-of-view of entire skeleton. The tracer technetium-99m-labeled diphosphonates (99mTc-MDP) is the most widely used bone agent, providing excellent contrast between normal and diseased bone. In general, uptake of the tracer depends on local blood flow, osteoblastic activity and extraction efficiency (Gnanasegaran *et al.*, 2009; O'Connor *et al.*, 1991), which can reflect early metabolic changes often several weeks or even months before apparent morphological changes on conventional radiological images (Van den Wyngaert *et al.*, 2016). Recently, bone scintigraphy accounts for more than 60% single photon imaging in China (about 1.586 million cases per year in 2020) (Jing and Sijin, 2020). Many busy nuclear medicine physicians already devote much of their time to handle a high volume of bone scintigraphy. However, due to the limitation of relatively lower resolution and visual contrast, bone scintigraphy interpretation is susceptible to subjective factors, such as experience of physicians. This may considerably affect the accuracy and repeatability of lesion detection and anatomical localization. The use of image processing methods can help improve efficiency and overall healthcare services. Nowadays, many computer-aided diagnosis systems have been developed to detect and segment hot spots (Chang *et al.*, 2011; Hojjatoleslami and Kittler, 1998; Huang *et al.*, 2007; Soloway *et al.*, 1988; Yin and Chiu, 2004).

For lesion detection, most of the proposed methods (Šajn *et al.*, 2007; Shiraishi *et al.*, 2007) focus on detection of hot spots rather than accurate segmentation, which results in poor interpretability. Adaptive threshold and region growing are two most commonly used approaches in segmentation. Obviously, due to weak boundary contrast and low signal noise ratio of bone scintigraphy, these two methods will not achieve satisfactory segmentation results. Sadik *et al.* (2006, 2008, 2009) presented several algorithms which addressed skeleton segmentation, hot spot detection and classification of bone scan examinations. Researchers in Sadik *et al.* (2009) used adaptive threshold of a specific region for hot spot segmentation, demonstrated the ability of segmentation in lesion detection. Apiparakoon *et al.* (2020) build a neural network to segment lesion from bone scintigraphy images. It is worth mentioning that the aforementioned method was focused on the chest image instead of the whole body.

As for skeleton segmentation, previous studies outputted polygonal regions, which roughly approximated bone regions (Huang *et al.*, 2007; Yin and Chiu, 2004). Although the skeleton segmentation performance in the previous study (Sadik *et al.*, 2006) was improved (Sadik *et al.*, 2008) by techniques such as the multi-atlas segmentation (MAS), it was found to be sensitive to the initial position of the model and image noise. In addition, the whole skeleton was only divided into four large parts, each of which included several different bones. The spatial granularity of that is much coarser than needed for physician's reports. Some of the aforementioned problems have been solved using the atlas-based approach (Kikuchi *et al.*, 2012). There, a manually segmented atlas consisting of more than 10 bones was non-linearly registered to an input image, and the deformed labels were transferred to the image. The atlas-based segmentation is a promising approach, but it suffers from the problems of initial positioning of the atlas and geometrical typicality among the atlas and skeleton of an input image. The use of multiple atlas, instead of one, could partially alleviated these problems (Iglesias and Sabuncu, 2015).

The purpose of this study was to develop a completely automated method, based on image processing techniques and deep learning model, for multi-tasks of lesion detection and anatomical localization in whole-body bone scintigraphy.

## 2 Materials and methods

### 2.1 Dataset and pre-processing

A total of 617 patients from April 15th, 2019 to May 29th, 2020 with suspected bone metastasis and underwent whole-body bone scan were retrospectively analyzed. The studies involving human participants were reviewed and approved by Institutional Review Board of the Cancer Hospital & Shenzhen Hospital, Chinese Academy of Medical Sciences and Peking Union Medical College Union Hospital, and the requirement to obtain informed consent was waived.

The whole-body bone scans were obtained 3–4 h after an intravenous injection of 740–1110 MBq 99mTc-methylene diphosphonate (99mTc-MDP; purchased from Guangzhou HTA Co. Ltd.) with a γ-camera equipped with low-energy, high-resolution parallel-hole collimators (GE Discovery NM/CT 670 Pro; scan speed, 20 cm/min; matrix, 256 × 1024). DICOM images with 16 bit depth included both anterior and posterior views. The ground truth segmentation of lesion and skeleton were annotated by board certificated physicians.

The anterior and posterior images were divided into two groups and processed separately. Similar processing method was used in these two sets of images. Therefore, only the process of anterior image will be described below for simplicity.

### 2.2 Multi-task learning network

**Fig. 1.**
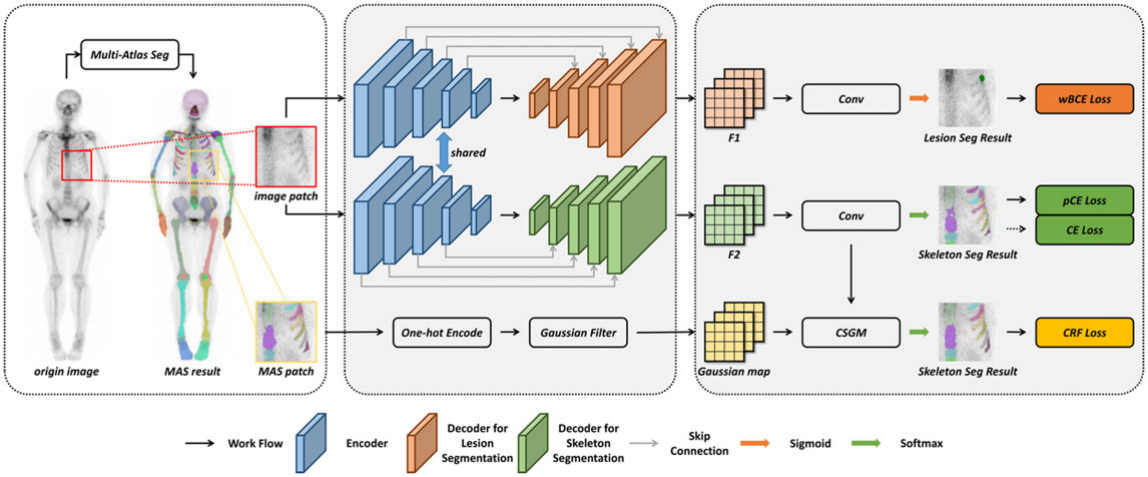
The overview of the proposed general framework. It composed of three parts. The first part uses the multi-atlas method to generate guidance information, which is followed by patch extraction. The second part is our multi-task network architecture, and the third part processes the feature map

A multi-task learning-based approach was employed to achieve high accuracy in skeleton segmentation and lesion extraction. The proposed network is shown in Figure 1. In it, we utilize U-Net as the backbone network for multi-task learning due to its great performance in biomedical image segmentation (Ronneberger *et al.*, 2015). Our architecture consists of three parts: (i) an encoding path, (ii) two decoding branch and (iii) skip connections between them. As shown in Figure 1, the first part generates pseudo ground truth for whole-body bone scan image by using MAS method. Then, we input the patch randomly extracted from the image and its corresponding MAS patch to the network. Denote the image patch as $I_{p}:\Omega \to R$ and the MAS patch as $S_{p}:\Omega \to R$. The network details were presented below.

#### 2.2.1 Encoder

Multitask learning algorithms incorporate the benefits from several related tasks to improve the overall performance by taking underlying common information that may be ignored by single task learning algorithms. Usually, proper pre-processing can facilitate segmentation. The gray-scale normalization was performed for image patch $I_{p}$ using $\hat{I_{p}}=I_{p}/255.0$ so that $\hat{I_{p}}$ was the normalized image patch.

The normalized image patch $\hat{I_{p}}$ was input to the encoder which employs several basic convolution blocks and four down-sampling operations to extract high-level semantic features. Then, high-level feature maps from encoding path were shared to extract common features for both lesion segmentation branch and skeleton segmentation branch.

#### 2.2.2 Decoder: whole-body bone scan lesion segmentation branch

In this branch, $\mathrm{Decode}r_{l}$ symmetrically utilized several basic convolution blocks and four up-sampling operations to restore the feature maps to the original input size and interprets the extracted features $F_{1}$ from the encoding path to predict the segmentation mask of the target. The skip-connection was used to introduce the feature information on the corresponding scale into the up-sampling process to provide multi-scale and multi-level information for later image segmentation. We added one convolutional layer and sigmoid function to the last layer of the $\mathrm{Decode}r_{l}$ to predict the lesion segmentation mask. This branch output a set of the corresponding lesion segmentation masks as $J_{p}:\Omega \to \left\{0,1\right\}$. The task of this branch was to learn a mapping F such that it gives an estimation of the mask $J_{p}$ given any image $I_{p}$:

$$\begin{array}{l} F\left(\hat{I_{p}}\right)=\hat{P}\left(J_{p}|\hat{I_{p}}\right)\\ =\hat{P}{\left(J_{p}\left(x\right)=1|\hat{I_{p}}\right)}_{x\in \Omega }\\ \approx P\left(J_{p}|\hat{I_{p}}\right)=J_{p} \end{array}$$

**Fig. 2.**
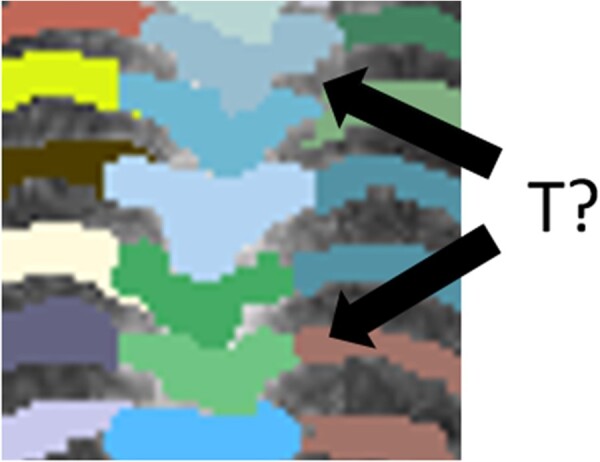
The challenges of patch-wise segmentation: mis-localization and classification of similar shaped structures. Organs with similar characteristics in close proximity are difficult to differentiate among each other. The figure shows an example of the thoracic portion in a posterior whole-body bone scintigraphy patch. ‘T?’ means the index of thoracic spine. It is difficult to distinguish the anatomy structure from this patch

One important challenge (Fig. 2) for patch based learning is how patches are selected from the entire image domain. If taken uniformly, then a large portion of the patches are likely to be just ‘background’, with their labels as all-zero masks. In practice, learning from such empty masks does more harms than wasting time. Indeed, it encourages the model to learn towards a trivial global optimal solution that maps every input to zero, especially when the area of the object takes a small portion of the entire image domain. Many researchers introduced extra mechanisms, such as using a bounding box to limit the area from where patches could be generated. However, how to determine such a bounding box during testing again poses new problems. Therefore, on one hand, we want the training patches to contain a certain amount of background patches to learn negative appearances. On the other hand, we do not want too many of them to steer the learning towards the trivial global optimal.

To address this challenge, the patches were obtained by sampling around the labeled area of the lesion. Specifically, the input ground truth segmentation mask was converted to a likelihood map l by Gaussian smoothing of the 0/1 mask. Then, rejection sampling was employed to sample from the l, which gives the patches within and around the target proportional to the likelihood of being inside the target. Doing this not only effectively balanced the distribution of the positive/negative patches in training but also accelerated the process especially when the target only takes a small portion of the entire image domain.

#### 2.2.3 Decoder: whole-body bone scan skeleton segmentation branch

In this work, each bone scan image is segmented into several different regions (56 for anterior and 68 for the posterior). This is to the best of our knowledge the most detailed anatomic segmentation. However, doing so significantly increases the annotation time (∼1.5 h/image).

Therefore, to balance the high-precision skeleton segmentation as well as the annotation expense, we opted to use the MAS method. This method usually requires only a small amount of annotation data. However, due to the noise of the bone scan image and the effect of the lesion on the registration effect, the accuracy of the segmentation is not sufficient. Although the number of atlases can theoretically be increased to improve the quality of segmentation, the annotation expense and the computational overhead will increase accordingly. In order to solve this challenging problem, we used the MAS method to generate pseudo ground truth, and then we used the generated label to guide and train the skeleton segmentation branch. Finally, we used a small amount of ground truth to refine and adjust the model.

In this branch, $\mathrm{Decode}r_{s}$ had the same architecture as $\mathrm{Decode}r_{l}$. The feature map $F_{2}$ was obtained by the $\mathrm{Decode}r_{s}$. $F_{2}$ and $S_{p}$ were input into the channel-spatial guidance module (CSGM). $S_{p}$ provided classification and positioning information for the segmentation network to predict the skeleton segmentation mask. Skip connections connected feature maps from the encoding path to the decoding path to propagate spatial information and refine segmentation outcomes. Given a set of corresponding skeleton segmentation masks by multi-atlas as $S_{p}:\Omega \to \left[0,C\right]$, the task of this branch was to learn a mapping G that estimates the mask S given any image $I_{p}$ and $S_{p}$:

$$\begin{array}{l} G\left(\hat{I_{p}},S_{p}\right)=\hat{P}{\left(S\left(x\right)|\left(\hat{I_{p}},S_{p}\right)\right)}_{x\in \Omega }\\ \approx P\left(S|\left(\hat{I_{p}},S_{p}\right)\right)=S. \end{array}$$

The individual components of the algorithm are detailed below.

#### 2.2.4 Channel-spatial guidance module

For utilizing the guidance information of the MAS, we designed a simple module behind the $\mathrm{Decode}r_{s}$. It took the feature map $F_{2}$ and the Gaussian map M of the MAS as input. Extracting the patches from the image could improve the accuracy of segmentation. But for some similar-shape structures, due to the lack of global information, segmentation often results in incorrect position and classification. We hope to introduce the results of MAS as the auxiliary information to guide the classification and position of the segmentation network, so as to obtain skeleton segmentation results. As a result, we designed a simple module named CSGM behind the $\mathrm{Decode}r_{s}$.

**Fig. 3.**
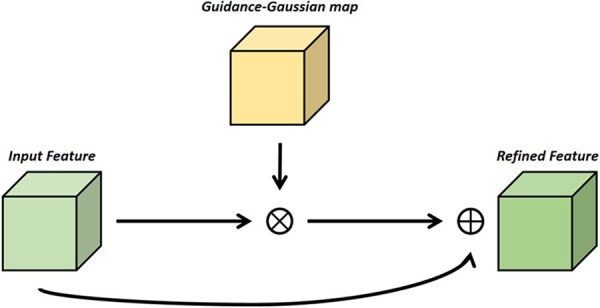
Channel-spatial guidance module

Given a feature map $F\in R^{C*H*W}$ and the MAS result patch $M\in R^{C*H*W}$ as input. The proposed module is shown in Figure 3. The overall process is:

$$F^{'}=F⊗f_{G}^{3*3}\left(M\right)+F,$$

where ⊗ denotes element-wise multiplication and $f_{G}^{3*3}$ represents a Gaussian filter operation with the filter size of 3*3. $F^{'}$ is the final refined output.

### 2.3 Multi-task loss function

During neural network training, the cost function is the key to adjusting a neural network's weights to create a better fitting machine learning model. Specifically, during forward propagation, the neural network takes training set data, and outputs are generated which in the case of classification indicate the probability or confidence in possible labels. These probabilities are compared to the target labels, and the loss function calculates a penalty for any deviation between the target label and the neural network's outputs. During back-propagation, the partial derivative of the loss function is calculated for each trainable weight of the neural network. The weights are adjusted by these partial derivatives. Under normal conditions, back-propagation iteratively adjusts the trainable weights of a neural network to produce a model with lower loss.

For a better explanation in the following parts, we defined some symbols and operations here. All training image patches were represented as x. We used y and z to represent the sets of pixels in lesion segmentation result and skeleton segmentation result according to the ground truth mask. F represented lesion segmentation sub-network, and G represented skeleton segmentation sub-network.

#### 2.3.1 Lesion segmentation

For the task of binary lesion segmentation, we usually used weighted binary cross-entropy (wBCE) as a loss function to supervise the neural network. Denoted the prediction as $\hat{y}=F\left(x\right)$. The standard weighted binary cross-entropy loss function is given by:

$$L_{\mathrm{wBCE}}\left(\hat{y},y\right)=-\left[w\times y\log\hat{y}+\left(1-y\right)\log\left(1-\hat{y}\right)\right],$$

where w is the weight balance parameter. The additional weight can be set to adjust the importance of the positive labels. A common use was to give more weight to lesion class.

#### 2.3.2 Skeleton segmentation

Training a neural network G for the task of segmentation required pairs of images x with their anatomical counterparts z, conveying per-pixel class assignments from C classes. Let us denote with N the total number of pixels in an image, positions of pixels with subscripts, and the probability of class i in the prediction $\hat{z}=F\left(x\right)$ as $\hat{z}\left(i\right)$. The conventional loss for multi-class classification problems is the cross-entropy loss, which takes the form

$$L_{CE}\left(\hat{z_{i}},z_{i}\right)=-\sum_{c=1}^{C}z_{i}\left(c\right)\log\hat{z_{i}}\left(c\right),$$

where z is given as a probability distribution, or

$$L_{CE}\left(\hat{z_{i}},z_{i}\right)=-\log\hat{z_{i}}\left(z_{i}\right),$$

where z takes on class labels.

Minimization of the regularized losses is a principled approach to weak supervision well-established in deep learning, in general. However, it is largely overlooked in semantic segmentation currently dominated by methods mimicking full supervision via ‘pseudo’ fully labeled masks (proposals) generated from available partial input. To obtain such full masks the typical methods explicitly use standard regularization techniques for ‘shallow’ segmentation, e.g. graph cuts or dense conditional random fields (CRFs). In contrast, we integrated such standard regularizers directly into the loss functions over partial input. This approach simplified weakly supervised training by avoiding extra CRF inference steps or layers explicitly generating full masks, while improving both the quality and efficiency of training.

In the fully supervised setting, nearly all pixels of the training images were annotated, forming a dense map of annotated pixel $m_{i}\in \left\{0,1\right\}$ of the same size as y, with possible unannotated exceptions including ambiguous or irrelevant classes. In weakly supervised setting, only a few pixels of the training images were annotated, forming a partial map of annotated pixel $m_{i}$, whose sparsity depends on the type of weak annotation.

Since the unannotated pixels present less information to the learning process, in particular those remote to the target, they are left out of consideration in the loss function which is used to compute gradients with back-propagation, i.e.

$$L_{\mathrm{pCE}}\left(\hat{z},z\right)=\frac{\sum_{i=1}^{N}m_{i}L_{CE}\left(\hat{z_{i}},z_{i}\right)}{\sum_{i=1}^{N}m_{i}}.$$

Note that, in the literature the cross-entropy loss for weakly supervised segmentation is usually called partial cross-entropy (pCE) due to the partial maps of annotated pixels $m_{i}$.

Since just applying pCE on weak annotations usually does not provide enough supervision signal close to semantic boundaries, it was natural to expect worse predictions where this supervision is unavailable. To alleviate that, we used the annotated inputs as seeds of supervision signal for pCE, and propagated it to the surrounding pixels with other forms of regularization. This approach had proven to work in both classical CRF post-processing (Krähenbühl and Koltun, 2011) as well as a loss function (Tang *et al.*, 2018). Yet, we were constraining our setting to a single round of joint training with no pre-/post-processing operations. The pairwise potential term in CRF energy function can be relaxed to a quadratic form which works for softmax output form the network:

$$L_{\mathrm{CRF}}=\sum_{ij}\sum_{c=1}^{C}\psi \left(y_{i}\left(c\right),y_{j}\left(c\right)\right)=\sum_{ij}\sum_{c=1}^{C}y_{i}\left(c\right)\left(1-y_{j}\left(c\right)\right)W_{ij},$$

where i, j are pixels, ψ denotes the pairwise potentials which used to describe the relationship between different pixels, and W is the affinity matrix between different pixels and is calculated by bilateral Gaussian filter. The total loss is expressed as:

L=LwBCE+LCE+LCRF, supervisionLwBCE+LpCE+LCRF, unsupervision

#### 2.3.3 Implementation

**Fig. 4.**
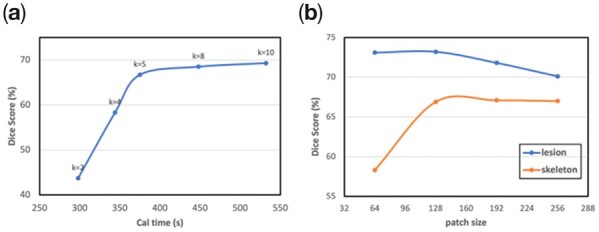
Experiment of the effect of model performance with hyperparameters

The proposed model was implemented in Python 3.7, PyTorch 1.6.1. Training and segmentation were conducted on NVIDIA V100 Tesla Graphics Processing Unit (GPUs). In the MAS, we take out half of images from the labeled training dataset for testing. Figure 4a below shows the computation time and mean dice similarity coefficient (mDSC) for different numbers of atlas. Usually, the more atlas is used, the better the segmentation result will be. However, as can be seen in Figure 4a, after five atlases, the improvement saturates but the calculation time keeps growing linearly. Since our purpose of using MAS is to obtain a reliable weakly labeled segmentation result, and then use the network to optimize the result. Therefore, considering the balance between calculation time and segmentation performance, the atlas number was set to 5. In each epoch, every input volume was randomly cropped into 100 128×128 patches. The results of different patch size on patchNet are shown in Figure 4b. The best results are obtained when the patch size is set to 128. Then, we used adaptive moment estimation optimization to train the network, the initial learning rate was $1\times 10^{-3}$ with a decay rate of $1\times 10^{-8}$ and batch size of 100. Weight decay and momentum were $1\times 10^{-4}$ and 0.9, respectively. The maximum number of epochs was set to 1000, and the optimal number of epochs was determined when the mDSC of the validation dataset reached the maximum. The dataset was separated into training, validation and testing data as listed in Table 1. In the skeleton segmentation task, each labeled data needs to be annotated with 124 Region of Interest (ROIs), which is a resource- and time-consuming task. We collected a total of 120 labeled data over 6 months. To better evaluate the performance of our model, we try to increase the number of testing dataset as much as possible. And the labeled data were randomly separated into 48 for training, 10 for validation and 62 for testing.

**Table 1.** The total bone scan cases for lesion and skeleton segmentation

Task	Type	Amount of cases	Amount of training cases	Amount of validation cases	Amount of testing cases
Skeleton segmentation	Labeled	120	48	10	62
	Unlabeled	497	497	0	0
Lesion segmentation	Labeled	617	545	10	62

## 3 Results

### 3.1 General characteristics of datasets

**Fig. 5.**
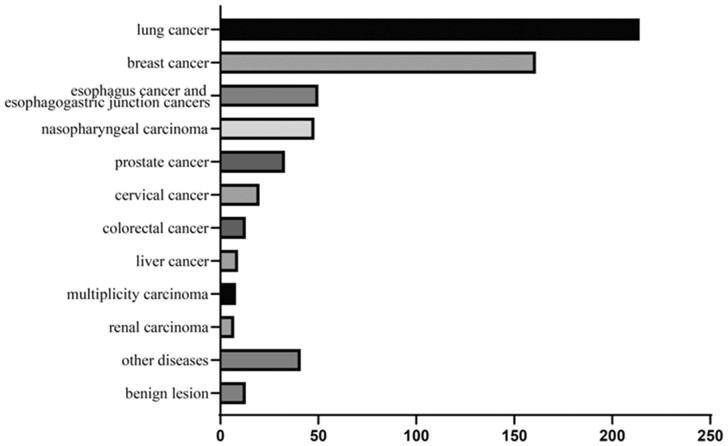
The summary of the datasets in terms of concerned disease types

Among the 617 cases, 301 (49%) were males and 316 (51%) were females. The average age was 52.7 ± 8.02 years (range 22–86 years). As shown in Figure 5, in terms of disease types, lung cancer (214, 34.7%) and breast cancer (161, 26.1%) accounted for the majority of cases, followed by esophagus cancer and esophagogastric junction cancers (50, 8.1%), nasopharyngeal carcinoma (48, 7.8%), prostate cancer (33, 5.3%), cervical cancer (20, 3.2%), colorectal cancer (13, 2.1%), benign(13, 2.1%), liver cancer (9, 1.5%) and renal carcinoma (7, 1.1%). In addition, there were eight cases (1.3%) of multiplicity carcinoma. A small number of other diseases, including five cases of head and neck cancers; four cases of uterine neoplasms, thyroid cancer, hypopharyngeal carcinoma and soft tissue sarcoma; three cases of ovarian cancer, cancer of the oral cavity and oropharynx cancer; two cases of gastric carcinoma and thymic carcinoma; one case of bladder cancer, vulvar cancer, pancreatic cancer, splenic angiosarcoma, laryngeal carcinoma, osteosarcoma and lymphoma.

### 3.2 Results of lesion segmentation and ablation studies

To demonstrate the effectiveness of different components in the lesion segmentation, we performed ablation studies to verify the effectiveness of the proposed patch-extracted strategy and multi-task learning strategy. We set traditional U-Net (Ronneberger *et al.*, 2015) as the benchmark in the ablation studies. Expected to learn more efficient information, the network we named patch-Net obtained the patches in special area from the bone scan image as input. Different from patch-Net, our network replaced the original encoder with the encoder that shares parameters with skeleton segmentation.

**Fig. 6.**
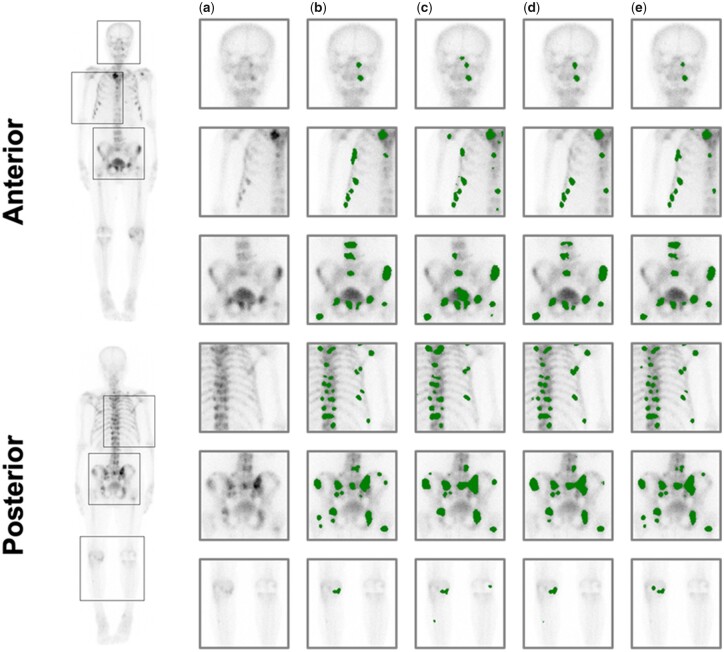
Comparison between each model and technique for lesion segmentation task in anterior and posterior. (**a**) Original images. (**b**) Expert annotation. (**c**) U-Net. (**d**) Patch-Net. (**e**) Ours

By applying U-Net, patch-Net and our method to the testing dataset, we can obtain different lesion segmentation results to compare the effect of each strategy. Typical segmentation results are visualized in Figure 6. As shown in Figure 6c, it is observed that the U-Net is incapable of achieving satisfactory segmentation results particularly for challenging cases with small sizes and irregular shapes. By obtaining the patches, patch-Net and our method shown better segmentation results which greatly improve the fineness of lesion segmentation and the accurate of lesion detection. They had higher sensitivity than U-Net, as shown in Figure 6d and e, this occasionally caused a false positive.

In addition, we performed statistical comparisons by collecting the mDSC, precision and recall values for different methods applied over the testing dataset. We can clearly observe that the patch-Net and our method performed superior to the U-Net (Table 2), which implies the effectiveness of the patch-extraction strategy. For a comprehensive comparison, we can also see that our method generally outperforms the patch-Net and achieved a higher statistical performance for mDSC and recall, indicating that the combination of both the patch-extraction strategy and multi-task learning strategy were effective in the lesion segmentation. Although our model has lower precision, for our application, recall is preferred over other metrics.

**Table 2.** Statistical comparisons of ablation studies in lesion segmentation

Pose	Model	mDSC	Precision	Recall
Anterior	U-Net	66.51	63.16 (703/1113)	73.08 (703/962)
	Patch-Net	73.22	**82.38 (837/1016)**	87.01 (837/962)
	Ours	**74.60**	80.07 (856/1069)	**88.98 (856/962)**
Posterior	U-Net	70.33	66.17 (837/1265)	78.89 (837/1061)
	Patch-Net	88.82	**83.85 (950/1133)**	89.54 (950/1061)
	Ours	**89.20**	81.32 (962/1183)	**90.67 (962/1061)**

Values in bold indicate the highest scores in the comparison experiment.

In order to assess the level of overfitting, we supplement a set of experiments for the lesion segmentation task. The dataset was divided with a ratio of 7:3 for 5-fold cross-validation. The experimental results are shown in Table 3. It can be seen that the average mDSC is close to previous experiments. This suggests that the overfitting phenomenon in the model is rather mild.

**Table 3.** Five-fold cross-validation experiment for lesion segmentation

	Fold-1	Fold-2	Fold-3	Fold-4	Fold-5	Mean ± std
mDSC	73.89	75.36	72.42	74.80	73.93	74.08 ± 1.00

### 3.3 Results of skeleton segmentation

To demonstrate the effectiveness of different components in the skeleton segmentation, we performed ablation studies to verify the effectiveness of the proposed patch-extracted strategy, semi-supervised strategy, CSGM module and multi-task learning strategy. We set traditional U-Net as the benchmark in the ablation studies. By applying them to the testing dataset, we can obtain different skeleton segmentation results. mDSC was taken to measure the skeleton segmentation performance based on the anatomical regions. As shown in Table 4, ‘Patch-input’ means the image patches are used as input. ‘Semi-supervised’ means the MAS result is as pseudo-label and the pCE loss and CRF loss are used as loss function. ‘CSGM’ means the MAS result is used as the channel and spatial guidance information for guiding the classification and position of the skeleton segmentation network. ‘Share encoder’ means replacing the original encoder with the encoder that shares parameters with lesion segmentation. As listed in Table 4, with these improvements, the performance has been increased to above 80%.

**Table 4.** Mean dice similarity coefficient (mDSC) of ablation studies on skeleton segmentation testing dataset

Inconsistency	UNet (mDCS, %)					
Patch-input		√	√	√	√	√
Semi-supervised			√		√	√
CSGM				√	√	√
Share encoder						√
Anterior	66.95	67.67	71.08	77.69	82.50	83.99
Posterior	68.52	66.15	69.41	76.33	80.90	81.55

We compare the proposed method with SOTA baselines on bone scan skeleton segmentation and semi-supervised learning. Specifically, SOTA baselines are MAS and Mean Teacher (Tarvainen and Valpola, 2017) model. Mean teacher is a classic semi-supervised learning method in medical image processing. U-Net is used as backbone and trained on labeled data as initial model in Mean Teacher experiment. As shown in Table 5, limited by the amount of labeled data, the accuracy was 3.39% lower than the baseline when we took the full image as input, only 0.41% higher than the baseline when patch-wise as input. A possible reason is ‘confirmation bias’ in the initial model. Confirmation bias also known as noise accumulation is deepened by training with false pseudo-labels provided by itself. This error accumulation is due to the pseudo-label method using its own predictions for supervision. Once the model predicts a sample incorrectly, this error will be treated as a supervisory signal, and this erroneous supervisory signal cannot be corrected by comparison with other samples.

**Table 5.** Mean dice similarity coefficient (mDSC) compared with SOTA on anterior skeleton segmentation testing dataset

Methods	mDSC (%)
U-Net	66.95
U-Net(patch-input)	67.67
Mean teacher	63.56
Mean teacher(patch-input)	68.08
MAS	76.31
Ours	83.99

**Fig. 7.**
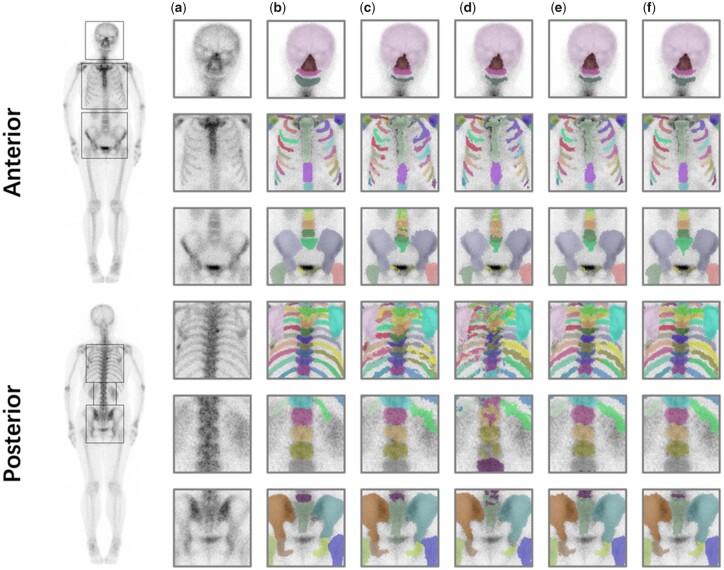
Comparison between each model for skeleton segmentation task in anterior and posterior. (**a**) Original images. (**b**) Expert annotation. (**c**) U-Net. (**d**) MAS. (**e**) Ours. (**f**) Ours (share Encoder)

Figure 7 presents the skeleton segmentation results on two poses in one patient using different methods. The first and second rows represent the original image and the ground truth on bone scan images respectively. Rows 3 through 6 show comparison of skeleton segmentation of the four representative methods where different colors were used to distinguish different regions. We can see that: (i) U-Net and patch U-Net are incapable to achieving satisfactory segmentation results particularly for challenging cases such as rib area and spine area, (ii) MAS yielded more accurate segmentation than corresponding networks without semi-supervised learning and (iii) our method achieved results which is visually indistinguishable from manual segmentation for almost all target regions.

## 4 Discussion

In this study, we proposed a multi-task convolutional neural network (CNN) architecture that integrates the whole-body bone scan lesion segmentation branch and skeleton segmentation branch. Through various experiments, we find that, in lesion segmentation, the proposed method has higher recall and mDSC, suggesting more robust performance on lesion detection and delineation. A total of 124 (56 for anterior and 68 for posterior) anatomical ROIs are extracted, which is to date the finest in the number of ROIs in the field. We proposed the CSGM which uses multi-atlas method to guide skeleton segmentation. Moreover, the proposed approach reliably balances unsupervised labels construction and supervised learning, providing insight for weakly labeled medical image analysis.

Through various experiments, we find that, in lesion segmentation, the proposed method is more robust on lesion detection and boundary delineation when the border of the lesion is of low contrast. Specifically, we designed a CSGM for the skeleton segmentation network and adopt the CRF loss function to refine the segmentation result. The effectiveness of this proposed method was evaluated on a dataset with 62 testing volumes.

Furthermore, experimental results demonstrated that the proposed multi-task model provided better results on both hot spot extraction and skeleton segmentation in comparison to the individually trained single-task models (Ronneberger *et al.*, 2015). Encoder with shared parameters was used to extract the underlying common feature between two segmentation tasks. Besides, misdiagnosis of small lesions also needs to be improved, which are subjected to the ongoing research. By integrating information into the segmentation flows through the joint training, our method performed best among them and achieved the highest score for two out of three metrics in lesion segmentation and the best mDSC in skeleton segmentation. In addition, the proposed approach that finds reliable unsupervised labels construction on unlabeled data to assist supervised learning provided insight for domains where the labeled data are limited, a typical situation in medical image processing.

The key issue the proposed method tried to address is the ‘small sample problem’. That is, we lack large volumes of detailed annotated images. Into that similar direction, other researchers have proposed various approaches. Soloway *et al.* (1988) proposed the extent of disease, which categorizes bone scan examinations into five grades based on the number of bone metastases. It is a straightforward idea but not suitable for detailed diagnosis. Yin and Chiu (2004) proposed a lesion extraction algorithm using the characteristic point-based fuzzy inference system. Huang *et al.* (2007) presented a bone scintigram segmentation algorithm followed by lesion extraction using adaptive thresholding with different cut-offs in different segmented regions. Chang *et al.* (2011) proposed an algorithm utilizing Gaussian function to approximate intensity probability distribution and perform hot spot segmentation via adaptive region growing (Hojjatoleslami and Kittler, 1998). Apiparakoon *et al.* (2020) used an instance segmentation network to detect lesions on chest. It is worth mentioning that training a ladder network required large amounts of data and poor results may be obtained when the dataset is small.

It is also noted that comparing different algorithms, each of which targets different body regions, diseases, datasets, etc., could be challenging. Firstly, the deep learning-based method mentioned in the previous paragraph focuses only on the chest region. Secondly, those studies have used internal datasets with their reference standard, and these datasets are not shared. Therefore, we made our best effort utilizing the data/open-sourced code and implementing the algorithms.

The current study has several limitations. Firstly, the retrospective nature of data collection from a single center may lead to sample bias. And the size of testing data in this study is relatively small and limited by the unlabeled data. Secondly, whether the trained model will work for other types of radiotracers is remained to be tested. While different tracers all show up as high uptakes (hot spots), their detailed texture information may vary and this may limit the generalization capability. Further investigation and prospective study are required to answer this clinically important question. Thirdly, lesion detection of the proposed method was designed only for hot spot segmentation, not including cold spots which are commonly due to osteolytic lesions or metallic objects. We should emphasize that our tool is not clinically validated and not certified, thus a visual control of the CNN results by experienced experts is necessary. We will further validate the generalization capability of the proposed semi-supervised structure through more experiments in future work.

## 5 Conclusion

We have developed and evaluated the use of a multi-task deep convolutional neural network that automated detects and locates lesions in bone scan images. By providing a MAS proposal before CNN architecture, the proposed model may be used for bone scan image interpretation on both hot spots extraction and skeleton localization.

## Supplementary Material

btac753_Supplementary_Data

## Data Availability

The data underlying this article cannot be shared publicly due to the privacy of individuals that participated in the study. The data will be shared on reasonable request to the corresponding author.
