# Supplementary material for: An end-to-end multi-task system of automatic lesion detection and anatomical localization in whole-body bone scintigraphy by deep learning
Source: Bioinformatics. 2022 Nov 23;39(1):btac753. doi: 10.1093/bioinformatics/btac753 (PMC9805554; doi:10.1093/bioinformatics/btac753)
Supplement: btac753_Supplementary_Data [file btac753_supplementary_data.docx]

**THE MULTI-ATLAS SEGMENTATION BASELINE**

Multi-atlas method is one of the widely used image segmentation techniques in biomedical applications. By utilizing the entire dataset of “atlases”, it has the flexibility to capture anatomical variation. As shown in Fig. 1, it first builds several representative images out of all the training images and perform multi-atlas segmentation with this new set of atlases. Then, several training images are independently registered to the novel image and the transformations are recorded. Finally, all the segmented label images are transformed and fused to form the final segmentation.

Specifically, denote the true skeleton segmentation of the template bone scan image $I_{T}$ as $S_{T}\left( x \right)$ and denote $A_{1}=\left( I_{T_{1}},S_{T_{1}} \right), \ldots, A_{n}=\left( I_{T_{n}},S_{T_{n}} \right)$, as $n$ atlases being considered. $I_{T_{i}}$ and $S_{T_{i}}$ are the $i^{\mathrm{th}}$ warped atlas images and the corresponding warped manual segmentation of this atlas, respectively. They are obtained by performing deformable image registration to the target image $I$.

The registration process of multi-atlas segmentation typically consists of pairwise multi-stage registrations between the bone scan image $I$ and each atlas template bone scan image $I_{T}$. It is commonly defined as the optimization problem over the parameters $\mu_{n}$ of the spatial transformation $T$, within a transformation space which maps each $x$ of image $I\left( x \right)$ to a location in image $I_{T}\left( x \right)$ by minimizing a cost function $C$

$$\hat{\mu_{n}}={\arg\min}_{\mu_{n}} C\left( I\left( x \right), I_{T}\left( T_{\mu_{n}}\left( x \right) \right) \right)$$

where $\hat{\mu_{n}}$ is the best parameters of the spatial transformation. The image registration strategies implementation was adapted from an open-source software toolbox Advanced Normalization Tools (ANTS) ^1^.

Each atlas produced one candidate segmentation for the target image. Each of the candidate segmentation may contain some segmentation errors. Label fusion is the process of integrating the candidate segmentation produced by all atlases to improve the segmentation accuracy in the final solution. Since all atlases shared common structures and similar appearances, independent errors in the final segmentation result were minimized following the joint label fusion scheme ^2^. With a measure of registration success for each transformed atlas, a weighted voting scheme achieved optimal label fusion that minimizes the expectation of combined label differences.

$$S\left( x \right)=\sum_{i=1}^{n} w_{i}\left( x \right)S_{i}\left( x \right)$$

Joint label fusion achieves consensus segmentation based on individual voting weights $w_{i}\left( x \right)$ and segmentation $S_{i}\left( x \right)$, such that

$$\sum_{i=1}^{n} w_{i}\left( x \right)=1$$

where weights were determined by

$$w_{x}=\frac{M_{x}^{-1}\cdot1_{n}}{1_{n}^{t}\cdot M_{x}^{-1}\cdot1_{n}}$$

where $1_{n}=\left[ 1,1,\ldots,1 \right]^{\top}$ is a vector of size $n$ and $M_{x}$ is the pairwise dependency matrix that estimates the likelihood of two atlases both producing imperfect segmentation on a per-pixel basis for the target image. The end result was a segmentation determined by the probability distribution of propagated labels provided by multi-atlas registrations.

**EVALUATION METRICS**

Our experiments were divided into two parts: lesion segmentation and skeleton segmentation. The overall performance of each model was assessed on the testing set, using the model which achieves the highest accuracy on the validation set.

We employed 4 metrics to evaluate the accuracy of our lesion segmentation, including Dice similarity coefficient (DSC), Jaccard similarity coefficient (JSC), precision and recall, which can be define as:

$$DSC=\frac{2\left| S\cap G \right|}{\left| S \right|+\left| G \right|}$$

$$JSC=\frac{\left| S\cap G \right|}{\left| S\cup G \right|}$$

where $S$ is estimated lesion segmentation result and $G$ is ground truth.

$$Precision=\frac{TP}{TP+FP}$$

$$Recall=\frac{TP}{TP+FN}$$

where TP is the number of lesions that are correctly predicted and labeled as positive whose JSC exceeds a threshold of 0.5 for each lesion. Conversely, FP (FN, resp.) is the number of lesions that are incorrectly predicted and labeled as positive (negative, resp.). The Dice similarity coefficient was utilized to evaluate the performance of lesions segmentation. Mean precision and mean recall were calculated to estimate the global lesion detection performance.

In skeleton segmentation, an instance segmentation was made for the complete bone scan and for the anatomical regions in Table 1. Let $s_{g}^{k}$, $s_{p}^{k}$ be the anatomical region of $k$ skeleton for ground truth and prediction, respectively. We quantified the overlapping for anatomical region $k$ using the DSC:

$$DSC\left( s_{g}^{k},s_{p}^{k} \right)=\frac{2\left| s_{g}^{k}\cap s_{p}^{k} \right|}{\left| s_{g}^{k} \right|+\left| s_{p}^{k} \right|}$$

Furthermore, we define the mean Dice similarity coefficient (mDSC) over all anatomical regions $k\in\left[ 1,K \right]$ as:

$$mDSC\left( s_{g},s_{p} \right)=\frac{1}{K}\sum_{k=1}^{K} DSC\left( s_{g}^{k},s_{p}^{k} \right)$$

where $K$ is the total number of anatomical regions. The mDSC between the segmented bone region and true region was computed to evaluate the performance of skeleton segmentation. Higher mDSC indicates more consistency between the ground truth and the segmentation results.

**REFERENCE**

1. Avants BB, Tustison N, Song G. Advanced normalization tools (ANTS). *Insight j*. 2009;2(365):1-35.

2. Wang H, Suh JW, Das SR, Pluta JB, Craige C, Yushkevich PA. Multi-atlas segmentation with joint label fusion. *IEEE transactions on pattern analysis and machine intelligence*. 2012;35(3):611-623.


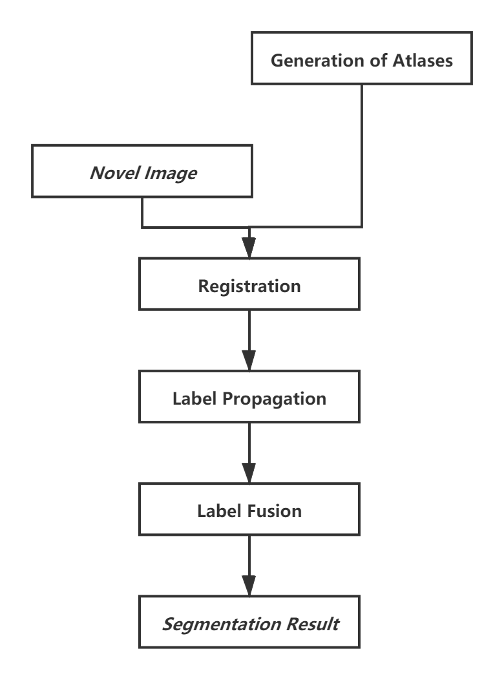


**Fig. 1** The structure of the Multi-Atlas segmentation.

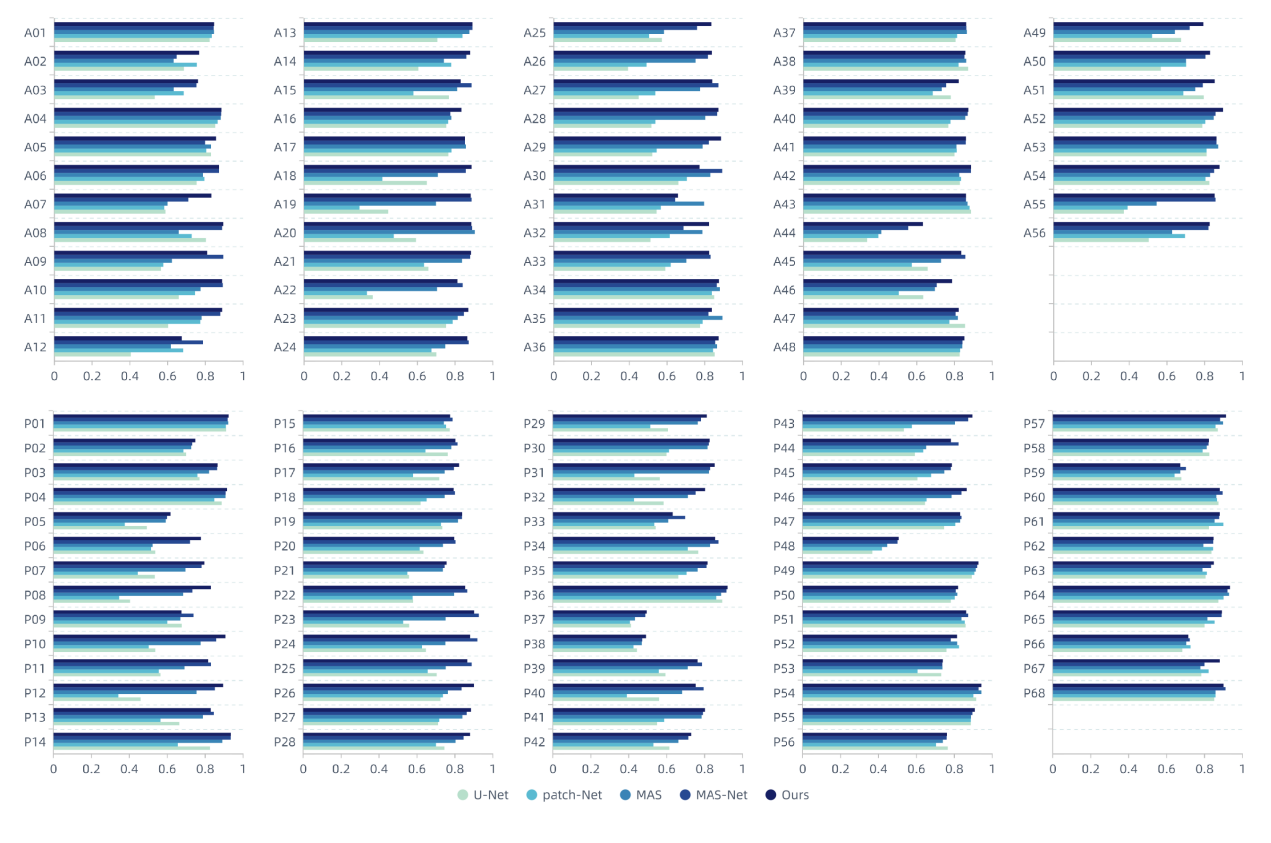


**Fig. 2** The mean DSCs of 124 anatomical regions (56 in anterior and 68 in posterior) based on 62 validation subjects, after segmentation by U-Net, patch-Net, MAS, MAS-Net and ours.


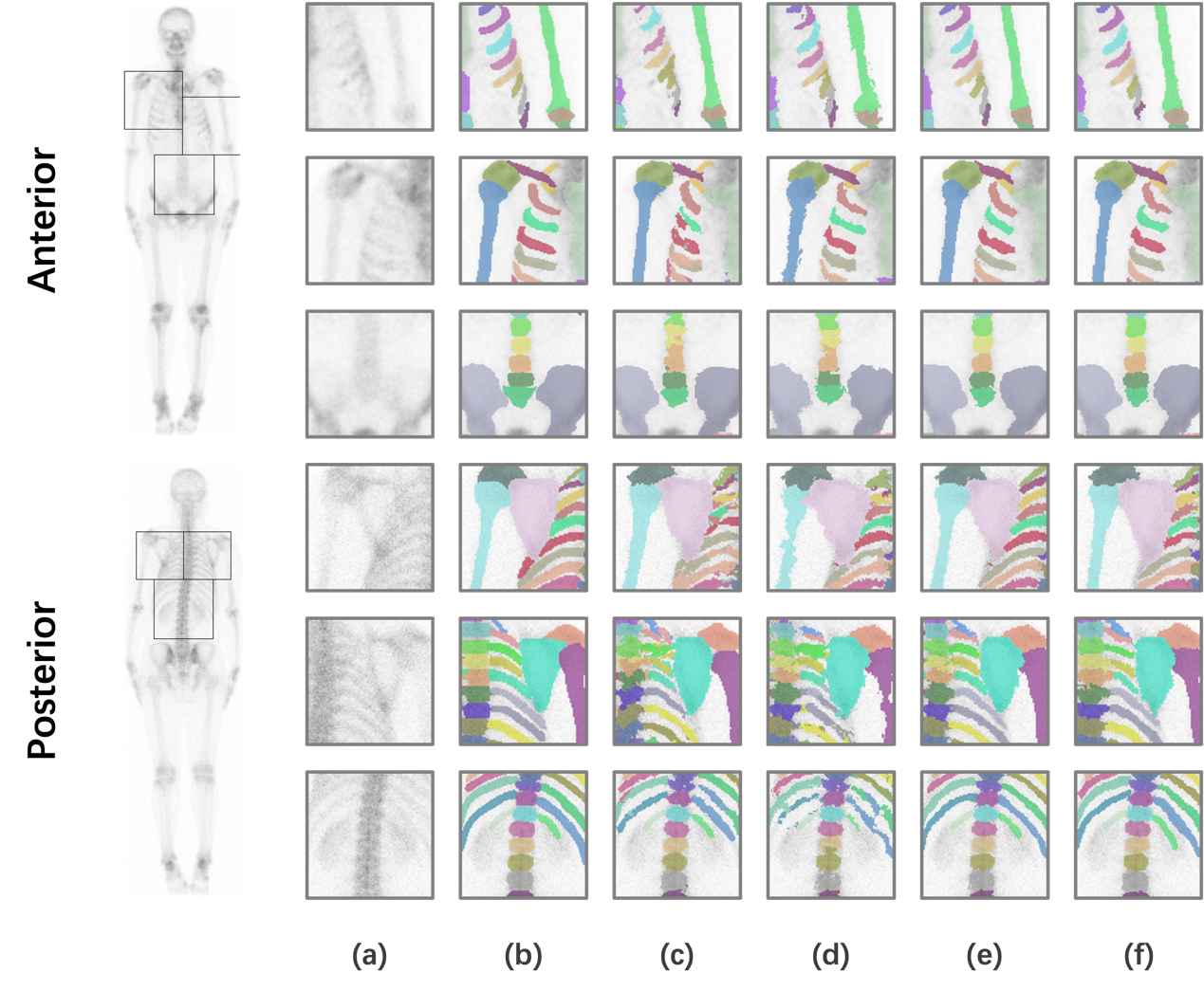


**Fig. 3** Comparison between each model for skeleton segmentation task in anterior and posterior. Case 1 of 3. (a)original images. (b)expert annotation. (c)U-Net. (d)MAS. (e)Ours. (f)Ours(share Encoder).


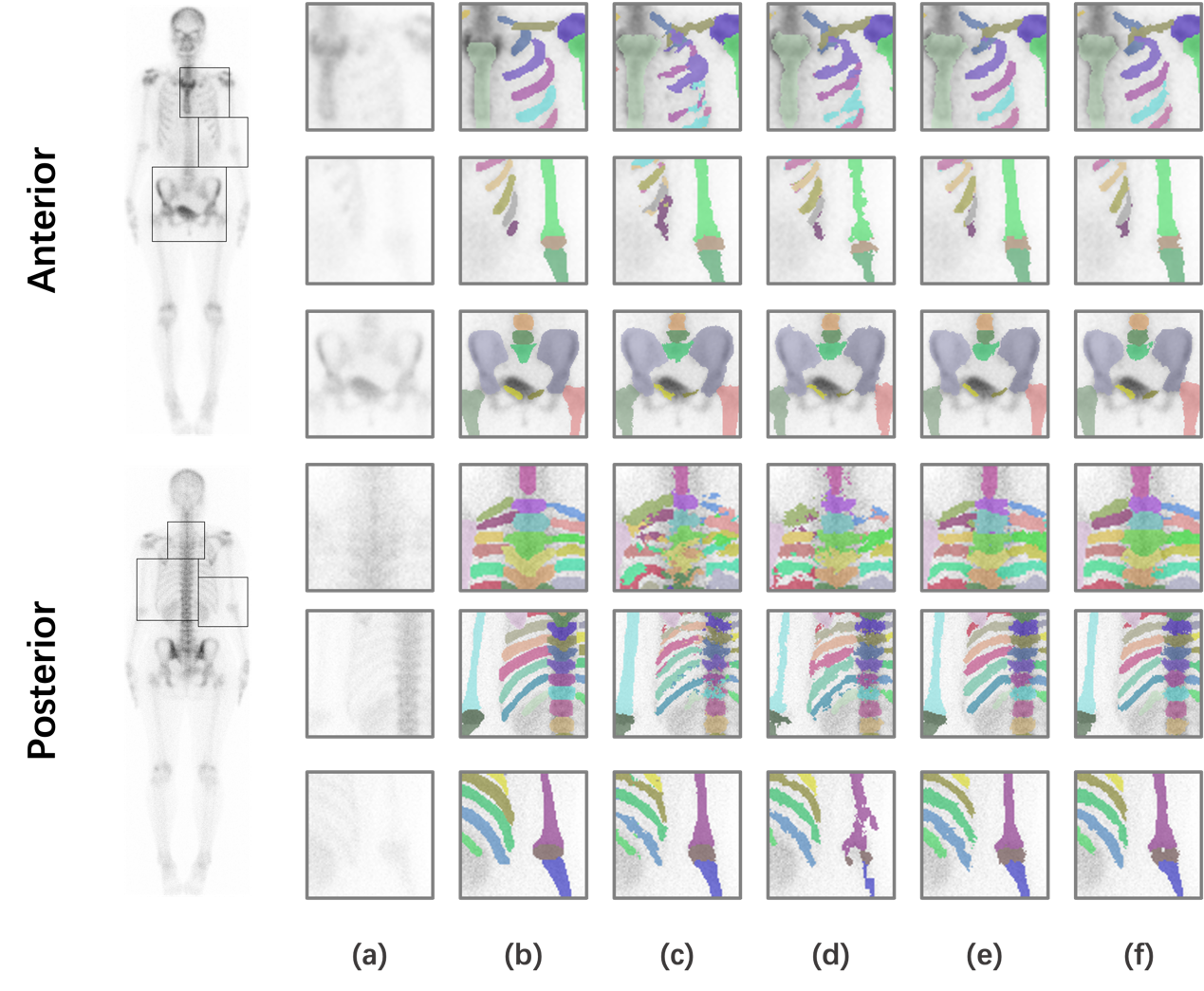


**Fig. 4** Comparison between each model for skeleton segmentation task in anterior and posterior. Case 2 of 3. (a)original images. (b)expert annotation. (c)U-Net. (d)MAS. (e)Ours. (f)Ours(share Encoder).


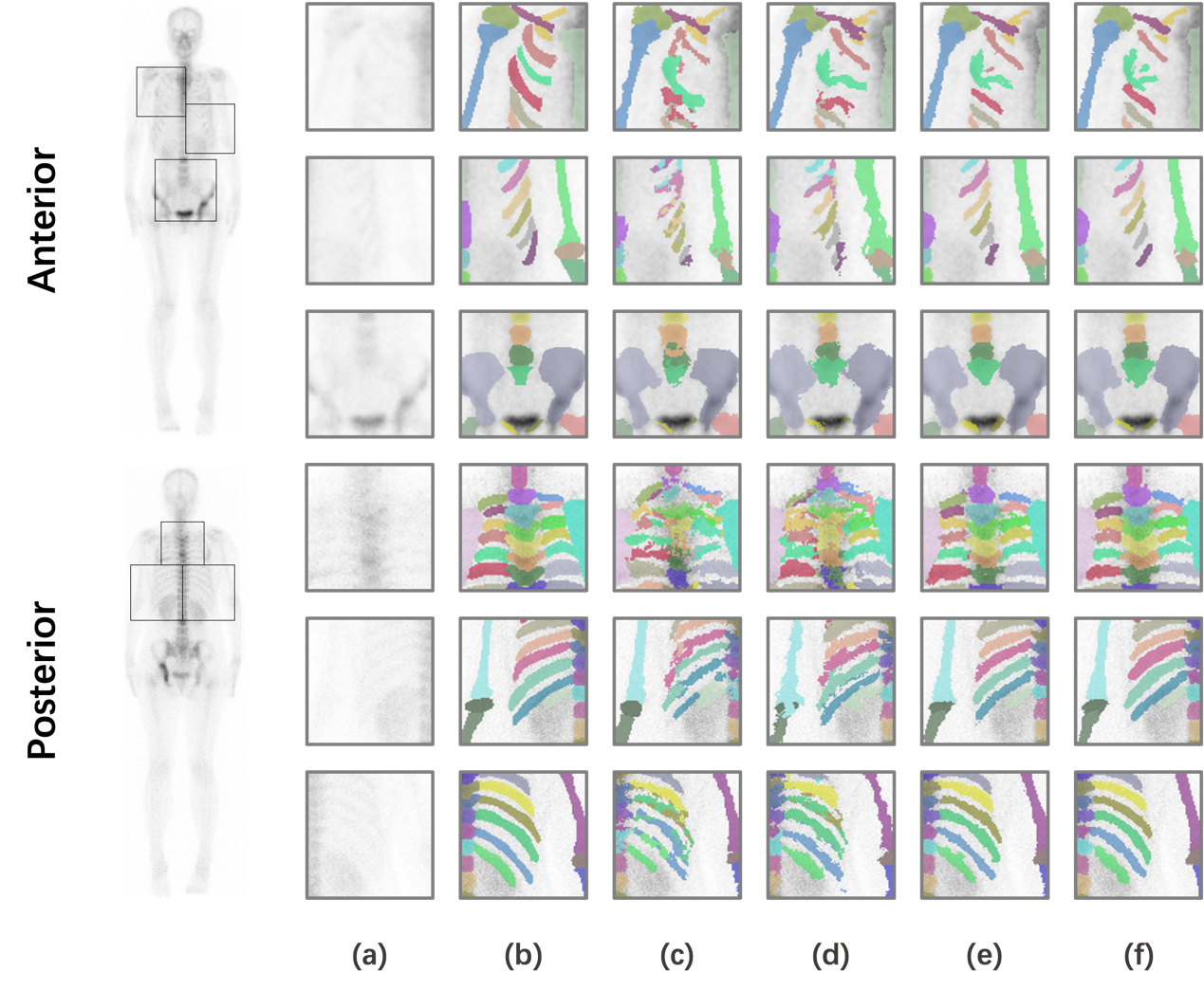


**Fig. 5** Comparison between each model for skeleton segmentation task in anterior and posterior. Case 3 of 3. (a)original images. (b)expert annotation. (c)U-Net. (d)MAS. (e)Ours. (f)Ours(share Encoder).

**Table 1** The name of the anatomical regions (L means left, R means right).

| ID | Fullname | ID | Fullname | ID | Fullname | ID | Fullname | ID | Fullname |
| --- | --- | --- | --- | --- | --- | --- | --- | --- | --- |
| A01 | NP | A13 | R 7th rib | A25 | L 1st rib | A37 | R foot | A49 | R elbow joint |
| A02 | Maxilla | A14 | R 8th rib | A26 | L 2nd rib | A38 | L femur | A50 | L elbow joint |
| A03 | Mandible | A15 | R 9th rib | A27 | L 3rd rib | A39 | L patella | A51 | R forearm |
| A04 | Skull | A16 | Sternum | A28 | L 4th rib | A40 | L tibia | A52 | L forearm |
| A05 | R shoulder | A17 | Thoracic vertebrae | A29 | L 5th rib | A41 | L foot | A53 | R hand |
| A06 | R clavicle | A18 | L1 | A30 | L 6th rib | A42 | R ilium | A54 | L hand |
| A07 | R 1st rib | A19 | L2 | A31 | L 7th rib | A43 | L ilium | A55 | R ankle |
| A08 | R 2nd rib | A20 | L3 | A32 | L 8th rib | A44 | R pubic | A56 | L ankle |
| A09 | R 3rd rib | A21 | L4 | A33 | L 9th rib | A45 | L pubic |  |  |
| A10 | R 4th rib | A22 | L5 | A34 | R femur | A46 | Sacrum |  |  |
| A11 | R 5th rib | A23 | L shoulder | A35 | R patella | A47 | R humerus |  |  |
| A12 | R 6th rib | A24 | L clavicle | A36 | R tibia | A48 | L humerus |  |  |
| P01 | Skull | P15 | L 11th rib | P29 | L1 | P43 | R 7th rib | P57 | R femur |
| P02 | Cervical vertebrae | P16 | L 12th rib | P30 | L2 | P44 | R 8th rib | P58 | R tibia |
| P03 | L shoulder | P17 | T1 | P31 | L3 | P45 | R 9th rib | P59 | R ankle |
| P04 | L shoulder blade | P18 | T2 | P32 | L4 | P46 | R 10th rib | P60 | R foot |
| P05 | L 1st rib | P19 | T3 | P33 | L5 | P47 | R 11th rib | P61 | L humerus |
| P06 | L 2nd rib | P20 | T4 | P34 | Sacrum | P48 | R 12th rib | P62 | L elbow joint |
| P07 | L 3rd rib | P21 | T5 | P35 | R shoulder | P49 | L ilium | P63 | L forearm |
| P08 | L 4th rib | P22 | T6 | P36 | R shoulder blade | P50 | L ischium | P64 | L hand |
| P09 | L 5th rib | P23 | T7 | P37 | R 1st rib | P51 | L femur | P65 | R humerus |
| P10 | L 6th rib | P24 | T8 | P38 | R 2nd rib | P52 | L tibia | P66 | R elbow joint |
| P11 | L 7th rib | P25 | T9 | P39 | R 3rd rib | P53 | L ankle | P67 | R forearm |
| P12 | L 8th rib | P26 | T10 | P40 | R 4th rib | P54 | L foot | P68 | R hand |
| P13 | L 9th rib | P27 | T11 | P41 | R 5th rib | P55 | R ilium |  |  |
| P14 | L 10th rib | P28 | T12 | P42 | R 6th rib | P56 | R ischium |  |  |

**Table 3** Mean Dice similarity coefficient (mDSC, %) of anatomical regions.

|  | Model | Area^[[1]](#footnote-1)^ | | | | |
| --- | --- | --- | --- | --- | --- | --- |
|  |  | Rib^[[2]](#footnote-2)^ | Spine^[[3]](#footnote-3)^ | Limbs^[[4]](#footnote-4)^ | Others | Total |
| **Anterior** | MAS | 73.90 (50.68-83.47) | 77.22 (65.06-84.53) | 79.10 (70.04-86.20) | 74.88 (63.48-87.81) | 76.31 (67.55-88.02) |
|  | U-Net | 58.15 (36.33-75.51) | 58.71 (37.10-78.94) | 75.11 (63.33-87.21) | 71.63 (58.82-85.13) | 66.95 (51.56-79.61) |
|  | Patch-Net | 63.40 (44.93-78.71) | 49.12 (36.33-59.17) | 74.42 (61.73-82.86) | 72.55 (59.31-86.32) | 67.67 (52.31-79.26) |
|  | MAS-Net | 82.67 (67.67-87.86) | 84.48 (65.26-90.45) | 83.05 (77.22-94.78) | 79.72 (71.05-89.17) | 82.50 (70.12-91.10) |
|  | Ours | ***82.99 (66.13-88.30)*** | ***85.60 (67.55-91.99)*** | ***85.17 (78.94-94.91)*** | ***82.59 (74.11-90.86)*** | ***83.99 (77.29-91.74)*** |
| **Posterior** | MAS | 69.15 (58.73-84.83) | 76.40 (60.08-85.92) | 81.85 (64.51-89.87) | 76.55 (60.10-85.53) | 76.27 (63.17-84.92) |
|  | U-Net | 57.87 (32.58-69.09) | 64.69 (55.73-76.35) | 81.05 (69.32-90.17) | 74.47 (57.45-82.24) | 68.52 (61.36-78.69) |
|  | Patch-Net | 54.03 (40.11-64.64) | 60.92 (52.33-74.86) | 81.62 (68.57-92.27) | 73.80 (61.15-86.41) | 66.15 (54.17-75.20) |
|  | MAS-Net | 75.78 (60.27-84.59) | 82.14 (57.56-90.47) | 83.88 (64.71-89.41) | 77.95 (65.32-90.53) | 80.90 (66.50-90.01) |
|  | Ours | ***76.45 (59.22-85.49)*** | ***82.65 (61.25-89.78)*** | ***84.67 (73.70-93.04)*** | ***78.57 (65.16-91.29)*** | ***81.55 (69.56-92.14)*** |

1. The name and ID for each region are provided as supplemental data in Table 1. [↑](#footnote-ref-1)
2. A7-A15, A25-A33 of anterior, P5-P16, P37-P48 of posterior [↑](#footnote-ref-2)
3. A17-A22, A46 of anterior, P2, P17-P34 of posterior [↑](#footnote-ref-3)
4. A34-A41, A47-A56 of anterior, P51-P54, P57-P68 of posterior [↑](#footnote-ref-4)
